# Supplementary figures and images for: A Comparative Analysis of Transcription Factor Expression during Metazoan Embryonic Development
Source: PLoS One. 2013 Jun 14;8(6):e66826. doi: 10.1371/journal.pone.0066826 (PMC3682979; doi:10.1371/journal.pone.0066826)

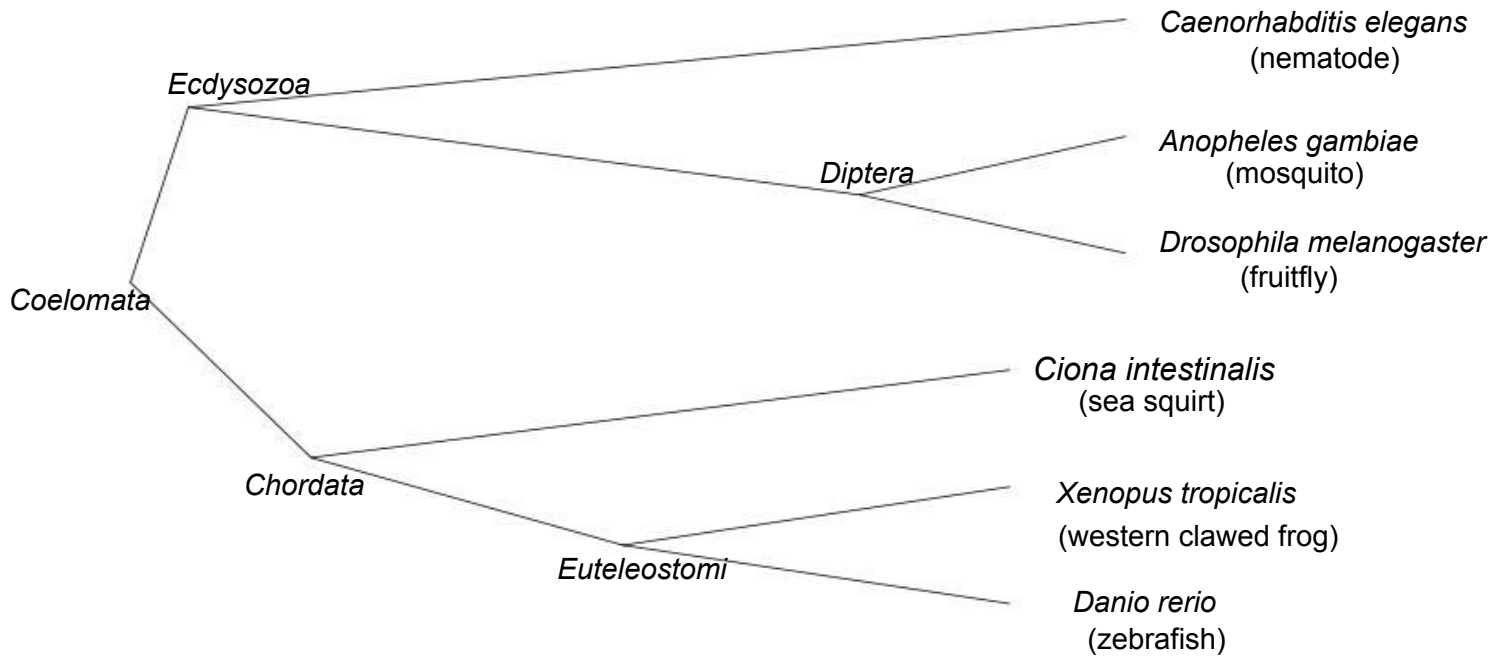

Supplement: Figure S1 — Evolutionary relationship between species considered in study. (PDF) [file pone.0066826.s001.pdf]

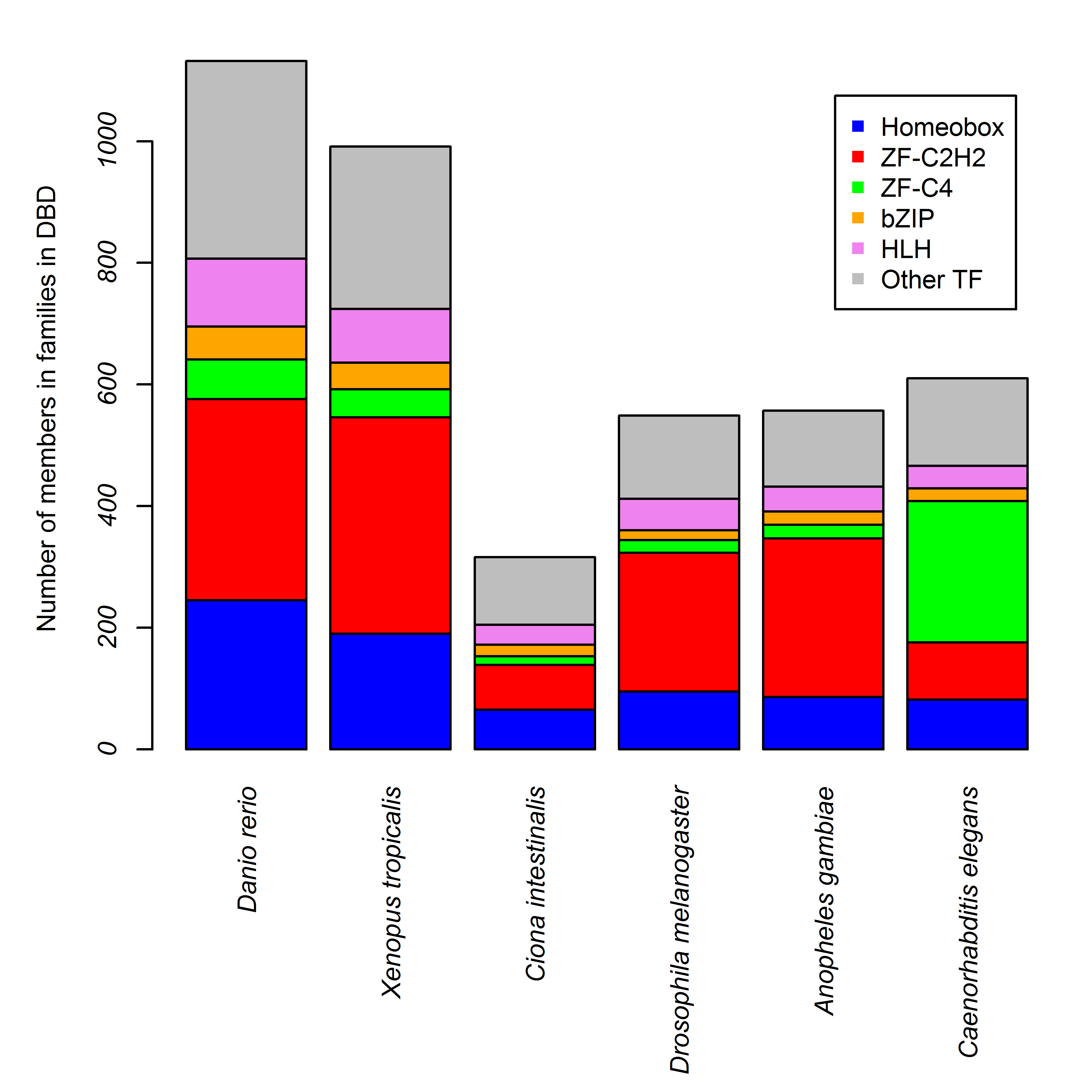

Supplement: Figure S2 — Distribution of TF families for TFs in DBD database for the species under consideration in this study. (TIF) [file pone.0066826.s002.tif]

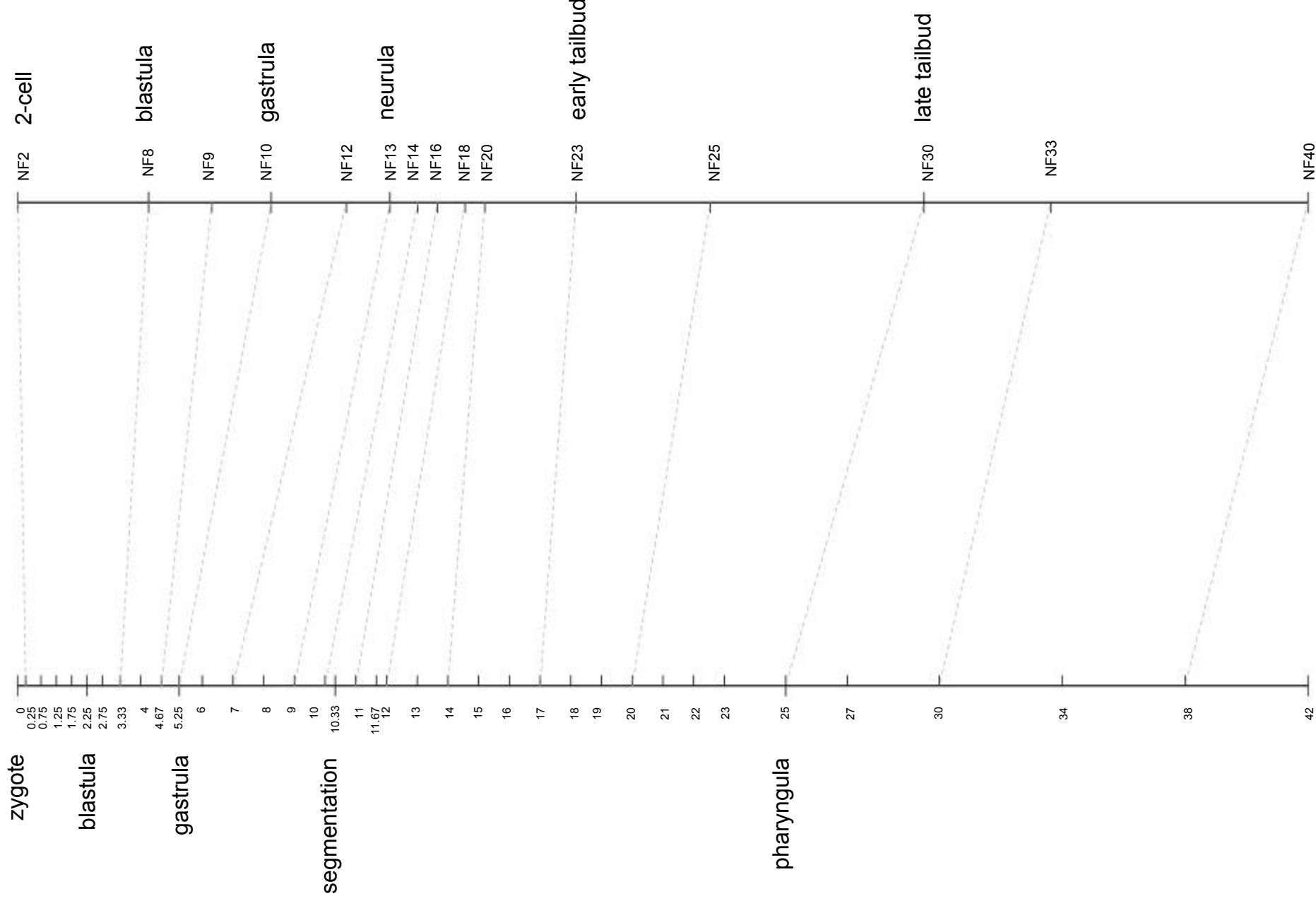

Supplement: Figure S3 — Time points for the zebrafish and Xenopus tropicalis microarray time courses. The gray dashed linesconnect time points in the two species that were considered as common time points. Spacing between stages for the Xenopus tropicalis timeline is based on approximate times between the onset of each stage. (PDF) [file pone.0066826.s003.pdf]
