# Supplementary material for: A Comparative Analysis of Transcription Factor Expression during Metazoan Embryonic Development
Source: PLoS One. 2013 Jun 14;8(6):e66826. doi: 10.1371/journal.pone.0066826 (PMC3682979; doi:10.1371/journal.pone.0066826)
Supplement: Table S1 — Counts of TFs and TF family members represented in each in situ hybridization dataset used for this study. Percentages in parentheses represent the percentage of TFs in the DBD database for the species covered in the dataset. (PDF) [file pone.0066826.s004.pdf]

| Species | Total TFs | Homeobox | ZF-C2H2 | ZF-C4 | HLH | bZIP |
|---|---|---|---|---|---|---|
| *Danio rerio* | 728 (64%) | 202 (82%) | 136 (42%) | 59 (91%) | 87 (78%) | 35 (65%) |
| *Ciona intestinalis* | 257 (81%) | 51 (78%) | 62 (84%) | 13 (93%) | 28 (85%) | 16 (84%) |
| *Drosophila melanogaster* | 452 (86%) | 74 (78%) | 194 (85%) | 16 (76%) | 45 (86%) | 15 (94%) |
| *Caenorhabditis elegans* | 252 (41%) | 51 (62%) | 26 (28%) | 59 (25%) | 19 (51%) | 8 (38%) |
