# Supplementary material for: A Comparative Analysis of Transcription Factor Expression during Metazoan Embryonic Development
Source: PLoS One. 2013 Jun 14;8(6):e66826. doi: 10.1371/journal.pone.0066826 (PMC3682979; doi:10.1371/journal.pone.0066826)
Supplement: Table S2 — Counts of TFs and TF family members represented in each microarray and RNA-Seq dataset used for this study. Percentages in parentheses represent the percentage of TFs in the DBD database for the species covered in the dataset. (PDF) [file pone.0066826.s005.pdf]

Species	Total TFs	Homeobox	ZF-C2H2	ZF-C4	HLH	bZIP
Danio rerio	917 (81%)	209 (85%)	244 (74%)	58 (89%)	91 (81%)	47 (87%)
Xenopus tropicalis	889 (90%)	184 (97%)	274 (78%)	45 (98%)	87 (99%)	44 (100%)
Drosophila melanogaster (array)	537 (98%)	94 (99%)	222 (97%)	21 (100%)	52 (100%)	16 (100%)
Drosophila melanogaster (RNA-Seq)	543 (99%)	94 (99%)	228 (100%)	21 (100%)	51 (98%)	15 (94%)
Anopheles gambiae	489 (88%)	78 (90%)	220 (84%)	20 (91%)	37 (90%)	18 (82%)
Caenorhabditis elegans	459 (75%)	66 (80%)	70 (74%)	184 (79%)	28 (76%)	12 (57%)
