# Supplementary material for: A Comparative Analysis of Transcription Factor Expression during Metazoan Embryonic Development
Source: PLoS One. 2013 Jun 14;8(6):e66826. doi: 10.1371/journal.pone.0066826 (PMC3682979; doi:10.1371/journal.pone.0066826)
Supplement: Table S3 — GO Terms significantly enriched in zebrafish clusters at p<0.01. (PDF) [file pone.0066826.s006.pdf]

Cluster	GO.ID	Term	All TFs	In Cluster	P-value
1	GO:0042074	cell migration involved in gastrulation	11	6	0.0029
2	GO:0007275	multicellular organismal development	323	45	0.0013
3	GO:0060788	ectodermal placode formation	13	6	0.0012
3	GO:0035121	tail morphogenesis	3	3	0.0013
5	GO:0007275	multicellular organismal development	323	94	2.6e-14
5	GO:0043010	camera-type eye development	38	16	0.0052
5	GO:0006355	regulation of transcription, DNA-depende...	612	119	0.0066
7	GO:0007165	signal transduction	133	27	0.0088
7	GO:0043401	steroid hormone mediated signaling pathw...	56	14	0.0089
9	GO:0043524	negative regulation of neuron apoptosis	2	1	0.0086
