# Supplementary material for: A Comparative Analysis of Transcription Factor Expression during Metazoan Embryonic Development
Source: PLoS One. 2013 Jun 14;8(6):e66826. doi: 10.1371/journal.pone.0066826 (PMC3682979; doi:10.1371/journal.pone.0066826)
Supplement: Table S4 — GO Terms significantly enriched in Xenopus tropicalis clusters at p<0.01. (PDF) [file pone.0066826.s007.pdf]

Cluster	GO.ID	Term	All TFs	In Cluster	P-value
1	GO:0016573	histone acetylation	4	3	0.0059
2	GO:0007067	mitosis	2	2	0.0071
2	GO:0051301	cell division	4	2	0.0381
4	GO:0045944	positive regulation of transcription fro...	20	10	1.1e-05
4	GO:0009952	anterior/posterior pattern specification	22	14	0.00035
4	GO:0014034	neural crest cell fate commitment	8	5	0.00061
4	GO:0045665	negative regulation of neuron differenti...	3	3	0.00131
4	GO:0030902	hindbrain development	11	8	0.00143
4	GO:0001756	somitogenesis	7	5	0.00166
4	GO:0030917	midbrain-hindbrain boundary development	6	4	0.00175
4	GO:0045666	positive regulation of neuron differenti...	6	4	0.00175
4	GO:0051090	regulation of sequence-specific DNA bind...	14	6	0.00376
4	GO:0042472	inner ear morphogenesis	13	7	0.00420
4	GO:0033504	floor plate development	6	4	0.00465
4	GO:0006916	anti-apoptosis	4	3	0.00481
4	GO:0000122	negative regulation of transcription fro...	17	6	0.00686
4	GO:0043049	otic placode formation	8	4	0.00686
5	GO:0007275	multicellular organismal development	192	56	3.5e-11
