# Supplementary material for: A Comparative Analysis of Transcription Factor Expression during Metazoan Embryonic Development
Source: PLoS One. 2013 Jun 14;8(6):e66826. doi: 10.1371/journal.pone.0066826 (PMC3682979; doi:10.1371/journal.pone.0066826)
Supplement: Table S5 — GO Terms significantly enriched in worm clusters at p<0.01. (PDF) [file pone.0066826.s008.pdf]

Cluster	GO.ID	Term	All TFs	In Cluster	P-value
9	GO:0016048	detection of temperature stimulus	1	1	0.0059
