# Supplementary material for: A Comparative Analysis of Transcription Factor Expression during Metazoan Embryonic Development
Source: PLoS One. 2013 Jun 14;8(6):e66826. doi: 10.1371/journal.pone.0066826 (PMC3682979; doi:10.1371/journal.pone.0066826)
Supplement: Table S6 — GO Terms significantly enriched in mosquito clusters at p<0.01. (PDF) [file pone.0066826.s009.pdf]

Cluster	GO.ID	Term	All TFs	In Cluster	P-value
1	GO:0006978	DNA damage response, signal transduction...	2	2	0.0042
1	GO:0010165	response to X-ray	2	2	0.0042
1	GO:0010332	response to gamma radiation	2	2	0.0042
1	GO:0031571	mitotic cell cycle G1/S transition DNA d...	2	2	0.0042
1	GO:0034644	cellular response to UV	2	2	0.0042
1	GO:0042771	DNA damage response, signal transduction...	2	2	0.0042
1	GO:0043523	regulation of neuron apoptosis	2	2	0.0042
6	GO:0007179	transforming growth factor beta receptor...	3	3	0.00083
8	GO:0045944	positive regulation of transcription fro...	5	4	0.0068
